# Maternal food-derived signals oscillate in the fetal suprachiasmatic nucleus before its circadian clock develops

**DOI:** 10.1371/journal.pbio.3003404

**Published:** 2025-09-26

**Authors:** Martin Sládek, Pavel Houdek, Tomáš Čajka, Alena Sumová

**Affiliations:** 1 Laboratory of Biological Rhythms, Institute of Physiology of the Czech Academy of Sciences, Prague, Czech Republic; 2 Laboratory of Translational Metabolism, Institute of Physiology of the Czech Academy of Sciences, Prague, Czech Republic; Charité - Universitätsmedizin Berlin, GERMANY

## Abstract

The ontogenesis of the circadian clock in the suprachiasmatic nuclei of the hypothalamus (SCN) and its sensitivity to maternal signals are not fully understood. Here, we investigated the development of the clock in the rat SCN from the fetal to the postweaning period and identified rhythmic metabolic signals from the mother to the fetal SCN. We determined daily expression profiles of clock genes (*Per2*, *Nr1d1*, *Bmal1*) and clock- and metabolism-related genes (*Dbp*, *E4bp4*) and performed time-resolved analysis of the metabolome and lipidome in the SCN and plasma of 19-day-old embryos (E19) and 2-, 10-, 20-, and 28-day-old pups (P02–28). Our data show that rhythms in the expression of canonical clock genes are absent at E19 and develop gradually until P10, but the *Dbp* rhythm was still developing between P20 and P28. Expression of the metabolism-sensitive gene *E4bp4* and levels of essential amino acids and other metabolites supplied by maternal food are rhythmic in the fetal SCN, which is lost after birth at P02 and reappears later in the postnatal period. Maternal food-derived metabolites were also rhythmic in fetal plasma. The temporal coherence of the fetal SCN metabolome and lipidome declines markedly and its rhythmicity disappears immediately after birth. The results revealed previously unforeseen pathways by which the fetal SCN may receive rhythmic information from the mother before its clock develops.

## Abstract 2

When and how are circadian rhythms established during development? This study characterizes rhythmic metabolites in the developing rat suprachiasmatic nuclei (SCN), revealing that maternal metabolic signals oscillate in the fetal SCN before the endogenous clock becomes functional and offering a plausible mechanism for maternal entrainment of the developing clock.

## Introduction

The suprachiasmatic nuclei in the hypothalamus (SCN) have been extensively studied for decades as a brain region where the central circadian clock is located [1]. The self-autonomous clock is at the top of the circadian system hierarchy [2]. As such, it generates a circadian (about-a-day) signal that is entrained with the 24-hour solar cycles of light and darkness (reviewed in [3]). The signal organizes rhythms in behavioral, humoral, metabolic, and neural processes that can synchronize the subordinate oscillators in various cells in the body to attain appropriate phase according to their function (reviewed in [4, 5]). As a result, a large part of the tissue-specific transcriptome, proteome, and metabolome daily varies to anticipate the environmental cycles [6–8].

These rhythms are generated at the molecular level by a transcriptional and translational feedback loop (TTFL, reviewed in [9]) in individual cells. The clock mechanism results in the rhythmic expression of families of clock genes (such as *Per1-3*, *Cry1-2*, *Nr1d1-2/Rev-Erbα-β*, *Rora-c*, *E4bp4/Nfil3*, *Bmal1/Arntl*, *Clock*, and *Npas2*). The protein products of these genes serve as transcriptional activators or repressors that govern the expression of a wide range of downstream clock-controlled genes. While the clock genes are expressed not only in the SCN but also in most body cells, clock-controlled genes are often tissue-specific [10]. Some of them (e.g., *E4bp4/Nfil3*, *Dbp*) are sensitive to changes in the metabolic state [11, 12] and may thus provide a link connecting the circadian clock and metabolic cycles.

The circadian system develops gradually from the fetal period to the postnatal stage (reviewed in [13]), and the maternal SCN plays a role in coordinating the developing rhythms [14]. However, the time when the SCN clock begins to generate the oscillations and plays a role of the central clock remains a matter of debate [15]. The shallow rhythms in expression of clock genes in the SCN samples collected from the fetal brains over 24-hour period were first detected 1–2 days before birth (embryonic day E20–E21 in rats), but their amplitudes were very low [16] and increased progressively during the postnatal period [17, 18]. Interestingly, our recent transcriptomic study showed that before the rhythm in expression of clock genes is detectable, a large number of genes oscillates in the fetal SCN [19], albeit with low amplitude. The results demonstrated a rhythmic transcriptional activity of the fetal SCN in the absence of the functional clock and support the view that maternal signals [20] drive the rhythms and may thus substitute immature fetal clock to promote rhythmicity in the fetal SCN tissue.

The aim of this study is to ascertain whether such circadian rhythmicity can also be detected at the level of the metabolome and lipidome in the fetal SCN and whether these rhythms involve potential maternal signals. Earlier studies in rats detected day/night variation in metabolic activity in the fetal SCN measured by ^14^C-labeled deoxyglucose levels 3–4 days prior to birth (embryonic day E19 in rats) and showed that the day/night ratio increased by rising the daytime levels during fetal development from E19 to E21 [21, 22]. The acute induction of metabolic activity in the maternal SCN by photic stimulation was not reflected in a change in the fetal rhythm, which suggested an endogenous origin of the rhythm in fetal SCN [22]. Nevertheless, this study used only two time points as a proxy for the fetal circadian rhythm and the day/night difference at E19 was small. Another study was unable to detect the rhythm [23]. Since then, the topic has not been addressed despite the progress in the determination of the rhythmic metabolome profiles in the adult SCN [24–26] and information about the development of rhythms in metabolome and lipidome in the fetal SCN is still missing. To fill the gap, we used the metabolomic and lipidomic profiling of the rat SCN samples collected over a 24-hour interval at five developmental stages representing the main milestones of the circadian system development—E19 (immature fetal clock), P02 (early neonatal stage), P10 (SCN morphology completed), P20 (SCN development completed, beginning of weaning), and P28 (weaning completed, adolescence). To identify potential metabolic food intake-related targets that may provide communication from the mother to the fetal SCN, we analyzed fetal plasma (E19) and plasma from P02 to P28 rats. The data revealed dramatic changes in metabolic cycles over the SCN maturation and provided novel insight into potential role of the nonhumoral pathways in setting the phase of the fetal SCN clock.

## Results

### The molecular clock in the rat SCN does not operate by E19 and develops gradually in clock gene-specific manner to the late postnatal age

**Fig 1:**
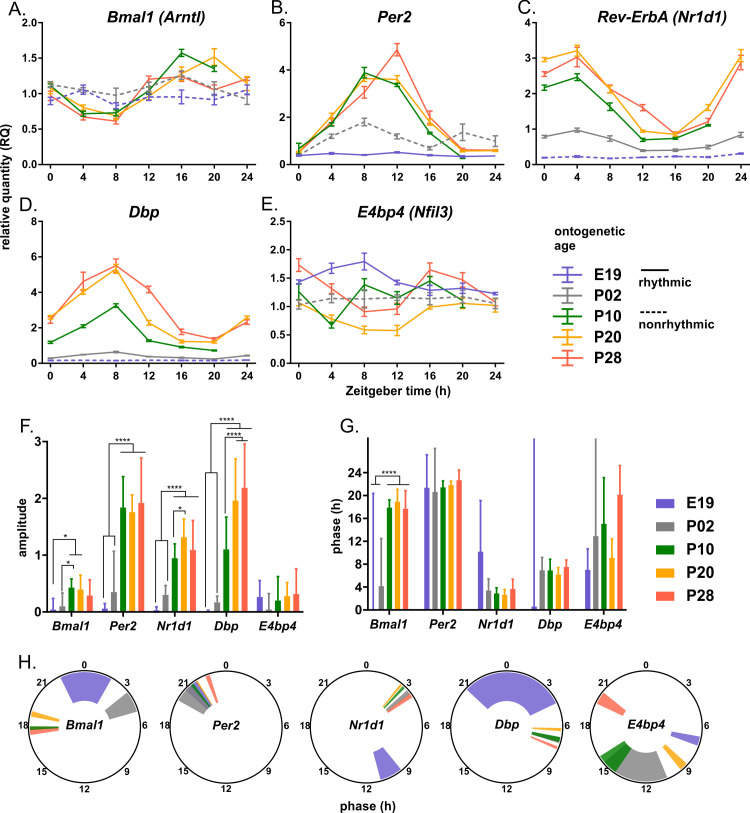
Daily expression profiles of rat clock genes in suprachiasmatic nuclei (SCN) across five developmental stages. SCN samples from embryonic age E19 (blue), postnatal ages P02 (gray), P10 (green), P20 (yellow), and P28 (red) were analyzed by RT qPCR with primers specific for **(A)** *Bmal1*, **(B)** *Per2*, **(C)** *Nr1d1*, **(D)** *Dbp*, and **(E)** *E4bp4*. Amplitude ± SD **(F)** and circadian phase ± SD **(G)** were determined by cosinor and analyzed by two-way ANOVA with Tukey’s multiple comparison test. **(H)** Circadian phase of each gene at the corresponding age was additionally visualized using polar coordinates, with the position of each bar representing the best-fit value and the width of each bar representing the standard error of the fit by cosinor. Rhythmicity was determined by eJTK; full or dashed lines depict the profiles that either did or did not pass the significance threshold (FDR-adjusted *P* < 0.05), respectively. Data expressed as mean ± SEM, *n* = 4–5/time point, *P < 0.05, ****P < 0.0001.

We analyzed expression of 3 clock genes (*Per2*, *Bmal1*, *Nr1d1*) and 2 clock controlled gene (*Dbp*, *E4bp4*) by RT qPCR in the SCN at E19, P02, P10, P20, and P28 (Fig 1). The profile was considered as rhythmic when both eJTK and the effect of time analyzed by 1-way ANOVA showed significant results (for results, see S1 Data). Comparison of the daily expression profile patterns (Fig 1A–1E) and the circadian parameters, such as amplitudes (Fig 1F) and phases (Fig 1G and 1H), shows that these expression rhythms develop gradually in a gene-specific manner.

The data show that the rhythmicity of *Bmal1*, *Nr1d1*, and *Dbp* expression is absent at E19. The *Bmal1* rhythm evolved to P10 by a decrease in expression levels during the daytime and their increase during the nighttime, as compared to the constitutive expression levels at E19 and P02. The amplitude of the other clock genes developed by increasing from the low expression levels during the daytime. Although *Per2* has an extremely shallow rhythm at E19, this could no longer be confirmed at P02, although expression increased. Importantly, the expression of *E4bp4* was significantly rhythmic at E19, with the opposite phase to the late postnatal stages, but expression levels decreased and the rhythm disappeared at P02. After birth at P02, both genes that were rhythmic at the fetal stage (*Per2* and *E4bp4*) lost their rhythms, *Bmal1* was still nonrhythmic, and the rhythms of *Per2*, *Nr1d1*, and *Dbp* showed only low amplitudes. By P10, the rhythms of *Bmal1*, *Per2*, and *Nr1d1* were fully developed, but the amplitude of the *Dbp* rhythm was still increasing between P10 and P20. The results show a gene-dependent gradual development of rhythmic expression profiles in the SCN clock. The rhythms in the expression of clock genes develop via increasing amplitudes by P10, and their phases do not change over development. However, the development of the rhythm of expression of the clock-controlled gene *Dbp* continues until P20. *E4bp4* is rhythmic during the fetal stage with opposite phase compared to adults; its rhythm is lost immediately after birth and reappears by P10.

### Complexity of the SCN lipidome and metabolome changes throughout development

To gain insight into the development of metabolism within the rat SCN, we conducted untargeted metabolomic and lipidomic profiling of SCN samples similarly collected from fetuses and pups as for the RT qPCR analysis, i.e., 5 replicate samples/time point (ZT0, 4, 8, 12, 16, 20, 24). We used reversed-phase liquid chromatography–mass spectrometry (RPLC–MS) in positive and negative ion mode for complex lipids, followed by the analysis of polar metabolites using RPLC–MS and hydrophilic interaction chromatography–mass spectrometry (HILIC–MS), each in positive and negative ion mode. Combining these 6 LC–MS platforms, we detected 485–513 unique metabolites in the SCN and 612–695 in plasma, depending on the developmental stage. The total number of imputed values for the SCN metabolites was 6,077 (representing 6.9% of all valid values, for age E19—6.09%, P02—5.98%, P10—6.2%, P20—6.88%, P28—9.37%). For PLS, it was 3,938 (3.4% of all valid values, E19—3.55%, P02—3.22%, P10—3%, P20—3.1%, P28—4.07%). The complete dataset is available as S1 Data.

**Fig 2:**
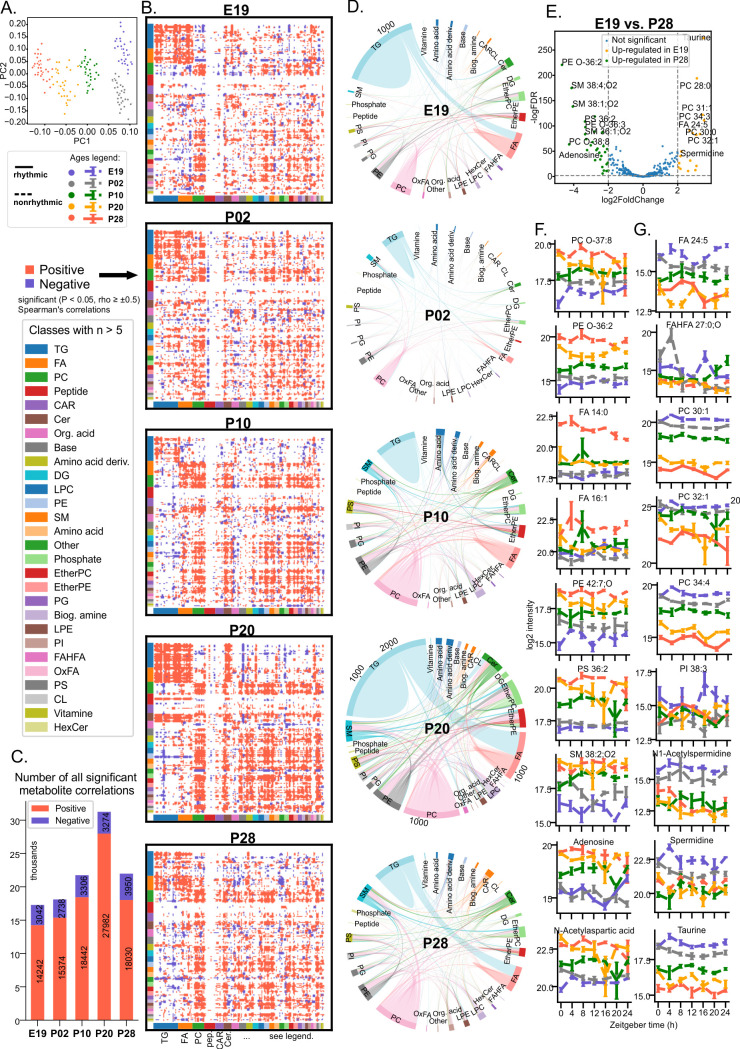
Coherence of SCN metabolic pathways changes during development. **(A)** Principal component analysis of all lipids and polar metabolites detected at E19–P28 shows a clear separation between ages. **(B)** Correlation Hinton plot for all detected lipids and polar metabolites in the E19–P28 SCN. Only the significant (|rho| ≥ 0.5, *P* < 0.05) Spearman’s correlations are depicted as either red (positive) or blue (negative) rectangles. Major classes are color coded and shown below the x-axis with selected examples next to the y-axis. **(C)** Number of all significant polar metabolite and lipid correlations (positive in red, negative in blue) across all analyzed ages. **(D)** Chord plots showing highly significant (*P* < 0.001) positive correlations (with the number of individual correlations illustrated by the thickness of a ribbon) between polar metabolites and individual lipids clustered to major classes within the developing SCN. Each link depicts Spearman’s correlation with rho ≥ 0.9. **(E)** Results of differential level (deseq2) analysis comparing levels of complex lipids and polar metabolites between E19 and P28. Only metabolites, which were detected at both compared ages, are shown. Examples of 24-hour profiles of compounds with gradually increasing **(F)** and decreasing **(G)** levels from E19 to P28. Embryonic age E19—blue, postnatal age P02—gray, P10—green, P20—yellow, and P28—red. Rhythmicity was determined by eJTK; full or dashed lines depict the profiles that either did or did not pass the significance threshold (FDR-adjusted *P* < 0.05), respectively. Data expressed as mean ± SEM, *n* = 4–5/time point. Lipid (sub)classes shortcuts: TG, triacylglycerols/triglycerides; FA, fatty acids; PC, phosphatidylcholines; CAR, acylcarnitines; Cer, ceramides; DG, diacylglycerols; LPC, lysophosphatidylcholines; PE, phosphatidylethanolamines; SM, sphingomyelins; EtherPC, ether-linked phosphatidylcholines; EtherPE, ether-linked phosphatidylethanolamines; PG, phosphatidyliglycerols; LPE, lysophosphatidylethanolamines; PI, phosphatidylinositols; FAHFA, fatty acyl esters of hydroxy fatty acids; OxFA, oxidized fatty acids; PS, phosphatidylserines; LPS, lysophosphatidylserines; CL, cardiolipins; HexCer, monohexosylceramides; CE, cholesteryl ester; EtherLPE, ether-linked lysophosphatidylethanolamines; NAE, N-acyl ethanolamines (endocannabinoids); NAorn, N-acyl ornithines.

Principal component analysis showed that ontogenetic age was the dominant factor determining the level of individual metabolites (Fig 2A), as the samples clustered together based on this factor, while their intragroup spread was comparable between ages. Lipids and polar metabolites grouped into distinct classes were then examined at each ontogenetic age for significant (Spearman’s rho ≥ 0.5, *P* < 0.05) correlations between their profiles, identifying those that may share metabolic pathways and function (Fig 2B). Triglycerides and fatty acids (FAs) showed a high number of positive correlations between each other throughout the development. Phosphatidylcholines were similarly highly positively correlated within their class, while peptides showed the lowest number of correlations within their groups and with other analyzed metabolites. However, the number of significant positive correlations between most metabolites increased gradually between E19 and P20, where it reached maximum (Fig 2C). Interestingly, after weaning at P28, the number of positive (but not negative) correlations decreased dramatically compared to P20. When focusing solely on the highest (rho ≥ 0.9) and most significant (*P* < 0.0001) positive correlations, the rewiring of the metabolic networks in the SCN after weaning is clearly visible, especially in the 3-fold decrease of correlated triglycerides and 2-fold decrease of correlated FAs (Fig 2D). Strikingly, there is a dramatic decrease in these highly correlated metabolites from the fetal SCN at E19 to early postnatal SCN at P02 (Fig 2D).

We performed differential level analysis (Fig 2E) comparing SCN metabolome and lipidome between E19 and P28, identifying significantly upregulated compounds at both developmental stages (only compounds detected at both E19 and P28 were visualized; for the list of all significantly different metabolites, see S1 Data). Strikingly, levels of a number of lipids and a few polar metabolites showed a progressive gradual increase or decrease during the ontogenesis. Examples of 30 identified compounds (S1 Fig) with gradually increasing levels from E19 to P28 (Fig 2F) include myristic (FA 14:0) and palmitoleic (FA 16:1) FAs, six ether-linked phosphatidylethanolamines (PE-O), seven sphingomyelins (SM), adenosine, and N-acetyl aspartate. Examples of 20 identified compounds (S2 Fig) with gradually decreasing levels from E19 to P28 (Fig 2G) include eight different phosphatidylcholines (PC), spermidine, *N*^1^-acetylspermidine, and taurine. Certain compounds occurred above detection level exclusively during prenatal (e.g., allantoin, thymidine), perinatal (e.g., isocitric acid), or postnatal period (e.g., serotonin), while others were detectable only before or after weaning (S3 Fig).

### Rhythms in metabolite and lipid levels disappear after birth and rapidly develop again after weaning

**Fig 3:**
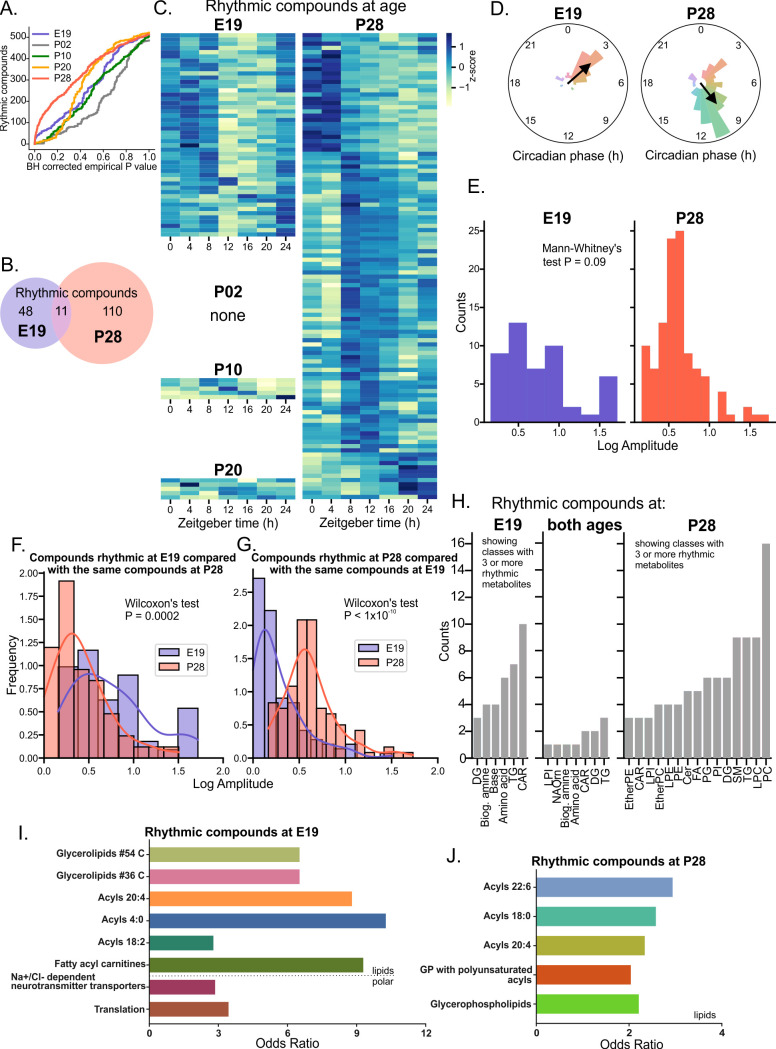
The amount of rhythmic polar metabolites and lipids in the SCN changes dramatically during development. **(A)** The rhythmicity threshold (FDR corrected empirical *P* < 0.05) was chosen after plotting the number of rhythmic compounds against *P* values of two detection methods (eJTK, one-way ANOVA). **(B)** Venn diagram of compounds identified as rhythmic in the E19 (blue) and P28 (red) SCN samples. **(C)** Heatmap of compounds identified as significantly rhythmic in the E19, P02, P10, P20, and P28 SCN, z-score normalized and sorted by phase. **(D)** Polar histogram of metabolites identified as rhythmic in the E19 (left) and P28 (right) SCN samples with calculated Rayleigh vector showing the mean phase. **(E)** Frequency histograms of amplitudes of metabolites identified as rhythmic in the E19 SCN (blue) and metabolites identified as rhythmic in the P28 SCN (red). **(F)** Frequency histograms of amplitudes of metabolites identified as rhythmic in the P28 SCN (red) and the corresponding metabolites (rhythmic or arrhythmic) in the E19 SCN (blue). **(G)** Frequency histograms of amplitudes of metabolites identified as rhythmic in the E19 SCN (blue, left) and of metabolites identified as rhythmic in the P28 SCN (red, right). **(H)** Polar metabolites and lipid classes (see Fig 2 for shortcuts) identified as rhythmic in the E19 (left, only classes with three or more rhythmic metabolites are shown), P28 (right), or at both ages (middle). Lipids and polar metabolites identified as rhythmic at E19 (I) or P28 (J) were subjected to overrepresentation analysis (ORA). Bar plots of significantly (*P* < 0.05) enriched lipid classes, lipid molecular species or Reactome pathways show corresponding odds ratios.

After examining the dependence of the number of rhythmic compounds on *P* value (Fig 3A), we set the rhythmicity threshold as Benjamini–Hochberg corrected empirical *P* values < 0.05. This identified 48 rhythmic compounds in the E19 SCN (9.5% of all detected compounds), no rhythmic compounds in the P02 SCN, 5 rhythmic compounds in the P10 SCN (1%), 5 rhythmic compounds in the P20 (1%), and 110 rhythmic compounds in the P28 SCN (21.6%), with 11 of those in common with E19 rhythmic group (Fig 3B and 3C). The results show a widespread shortfall of rhythmicity after the birth, likely connected with the loss of internal maternal signals, followed by a dramatic increase of circadian regulation of metabolism after the weaning, when the pups start to be fully dependent on ingestion of solid food.

The phase of the rhythmic compounds differed depending on the developmental stage, with most metabolites rhythmic at E19 clustering around ZT3 (Fig 3D, left), while the majority of compounds rhythmic at P28 clustering between ZT9–11 (Fig 3D, right). Interestingly, the overall amplitude of rhythmic metabolites did not differ significantly between E19 and P28 SCN (Mann–Whitney test, *P* = 0.09, Fig 3E). Moreover, Wilcoxon’s paired test showed that the metabolites that passed the rhythmicity threshold at E19 had significantly higher amplitude than the identical set of metabolites (11 of them rhythmic, the rest arrhythmic) at P28 (*P* = 0.0002, Fig 3F), and vice-versa, though that difference was much higher at P28 (Fig 3G, *P* < 0.0001).

### Distinct metabolites and lipid classes are rhythmic at different developmental stages

The composition of rhythmic compounds differed between E19 and P28 (Fig 3H), with fatty acylcarnitines and amino acids (AAs) being the most dominant rhythmic class at E19, while phosphatidylcholines, lysophosphatidylcholines, and sphingomyelins were dominant at P28. According to lipid enrichment analysis tool LORA that calculates ORA for lipidomics dataset and incorporates the annotation level and known information about the structures of lipid species (S4 Fig) [27], rhythmic lipids at E19 (Fig 3I) were highly enriched for fatty acylcarnitines (CAR, FA0707) class (FDR *P* < 0.0001), significantly enriched for acyl species (FA) 18:2 (FDR *P* = 0.0277), 20:4 and 2:0 (FDR *P* = 0.0317), and triacylglycerols with 54 (FDR *P* = 0.0377), and 36 (FDR *P* = 0.0464) carbons, respectively. The most over-represented lipid terms were CAR 20:4 and FA 20:4. Rhythmic lipids at P28 (Fig 3J) were highly enriched for glycerophospholipids (GP) category (FDR *P* = 0.0009), significantly enriched for GP species with acyls containing 2 or more double bonds (polyunsaturated, FDR *P* = 0.0405), and for acyl molecular species 18:0 (FDR *P* = 0.0128), 18:2 (FDR *P* = 0.0135), 20:4 (FDR *P* = 0.0128), and 22:6 (FDR *P* = 0.0128). The most over-represented lipid terms were phosphatidylinositols (PI) 18:0 and 20:4 and phosphatidyglycerols (PG) 18:0 and 20:4. Interestingly, both E19 and P28 rhythmic lipids were significantly enriched for Gene Ontology Biological Process term GO:0009410 (Response to xenobiotic stimulus, *P* = 0.0226), due to higher than predicted presence of triacylglycerols and sphingomyelins. According to WebGestalt ORA, rhythmic polar metabolites at E19 (Fig 3I) were enriched for Reactome pathways Translation (R-HSA-72766, *P* = 0.003, FDR *P* = 0.06) due to the presence of rhythmic AAs, and for Na^+^/Cl^−^ dependent neurotransmitter transporters (R-HSA-442660, *P* = 0.004, FDR *P* = 0.06). No significantly enriched polar metabolites were identified at P28.

**Fig 4:**
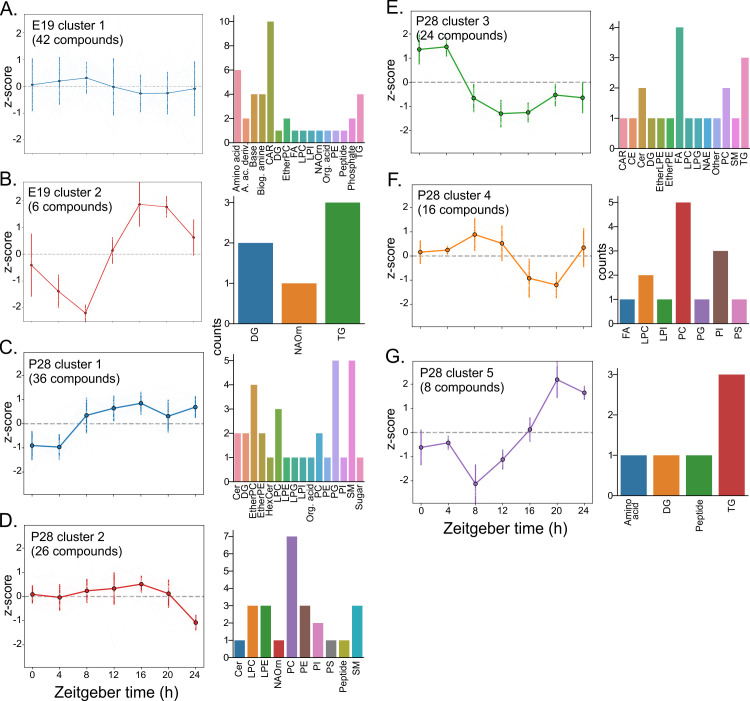
Rhythmic metabolites in the E19 SCN peak during the day. Majority of rhythmic lipids and polar metabolites in the E19 SCN peak during the day, while there are more diverse phase clusters in the developed P28 SCN postweaning. Polar metabolites and lipids identified as rhythmic in **(A, B)** E19 SCN (E19 clusters 1-2) or **(C–G)** P28 SCN (P28 clusters 1–5) were divided into subgroups based on hierarchical K-means clustering. Line plots on the left show traces of the normalized level of each rhythmic compound in each corresponding cluster. Bar plots on the right represent the lipid classes and polar metabolites in each rhythmic cluster. Lipid (sub)classes shortcuts: (TG, triacylglycerols/triglycerides; FA, fatty acids; PC, phosphatidylcholines; CAR, acylcarnitines; Cer, ceramides; DG, diacylglycerols; LPC, lysophosphatidylcholines; PE, phosphatidylethanolamines; SM, sphingomyelins; EtherPC, ether-linked phosphatidylcholines; EtherPE, ether-linked phosphatidylethanolamines; PG, phosphatidyliglycerols; LPE, lysophosphatidylethanolamines; PI, phosphatidylinositols; FAHFA, fatty acyl esters of hydroxy fatty acids (estolids); OxFA, oxidized fatty acids; PS, phosphatidylserines; LPS, lysophosphatidylserines; CL, cardiolipins; HexCer, monohexosylceramides; CE, cholesteryl esters; EtherLPE, ether-linked lysophosphatidylethanolamines; NAE, N-acyl ethanolamines (endocannabinoids); NAorn, N-acyl ornithines).

We used K-means clustering to separate rhythmic compounds in the E19 and P28 SCN into groups based on their circadian profiles. The vast majority of rhythmic metabolites in the E19 SCN peaked during the day (E19 cluster 1, Fig 4A), including all rhythmic AAs (for example, methionine), bases (e.g., cytidine) and carnitines. The second identified phase cluster at E19 peaked during the night and included only six lipid species (Fig 4B). After the rhythmic control of metabolism developed complexity after weaning, we were able to identify five different clusters with a diverse range of molecules (Fig 4C–4G). Interestingly, some metabolites were undetectable before P20 and gained rhythmicity only after weaning.

### Fetal SCN receives rhythmic metabolic signals from the mother before its clock starts to operate

Unexpectedly, the rhythms were much more developed in utero than in the first weeks after birth, suggesting that the rhythmic metabolism of the mother directly affects the developing fetal SCN. This was supported by detecting dietary polar metabolites and lipids that follow a circadian pattern with a distinct phase in the E19 SCN.

**Fig 5:**
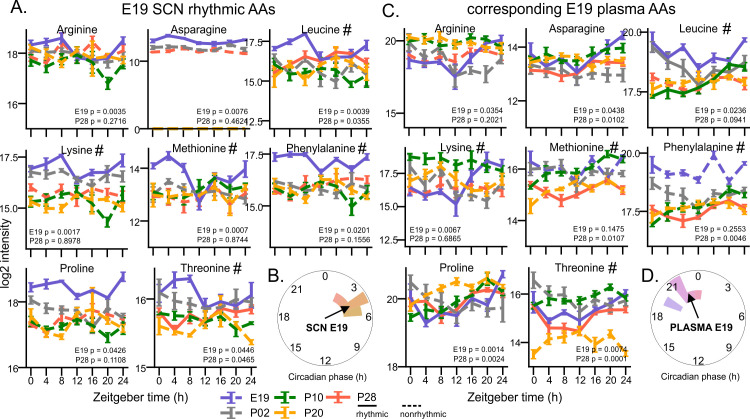
Essential amino acids are rhythmic in the E19 SCN. Amino acids (AAs, essential AAs marked by #) with rhythmic levels at E19 SCN are phase delayed compared to their rhythmic profiles in the E19 plasma. **(A)** Circadian time profiles of AAs identified as rhythmic at E19 (blue) SCN, with corresponding traces at P02 (gray), P10 (green), P20 (orange), and P28 (red) SCN. **(B)** Polar histogram of the corresponding AAs in the E19 SCN with calculated Rayleigh vector showing the mean phase. Corresponding time profiles **(C)** and polar histogram **(D)** of the same AAs in plasma. Rhythmicity was determined by eJTK; full or dashed lines depict the profiles that either did or did not pass the significance threshold (FDR-adjusted *P* < 0.05), respectively. Mean ± **S.**E.M, *n* = 4–5/time point, annotated with eJTK FDR corrected empirical *P* values for E19 and P28 data.

We found 7–8 rhythmic AAs in the E19 SCN (Fig 5A, with threonine being below the ANOVA but above the eJTK rhythmicity threshold), and five of them (leucine, methionine, phenylalanine, threonine, and lysine) belong to essential amino acids (EAAs) which cannot be synthesized by mammalian cells and need to be supplied from the maternal diet. Their phase was tightly clustered together around ZT4 (Fig 5B), several hours after their peak in fetal plasma (Fig 5C and 5D). Only three of these AAs stayed rhythmic in the SCN after weaning.

**Fig 6:**
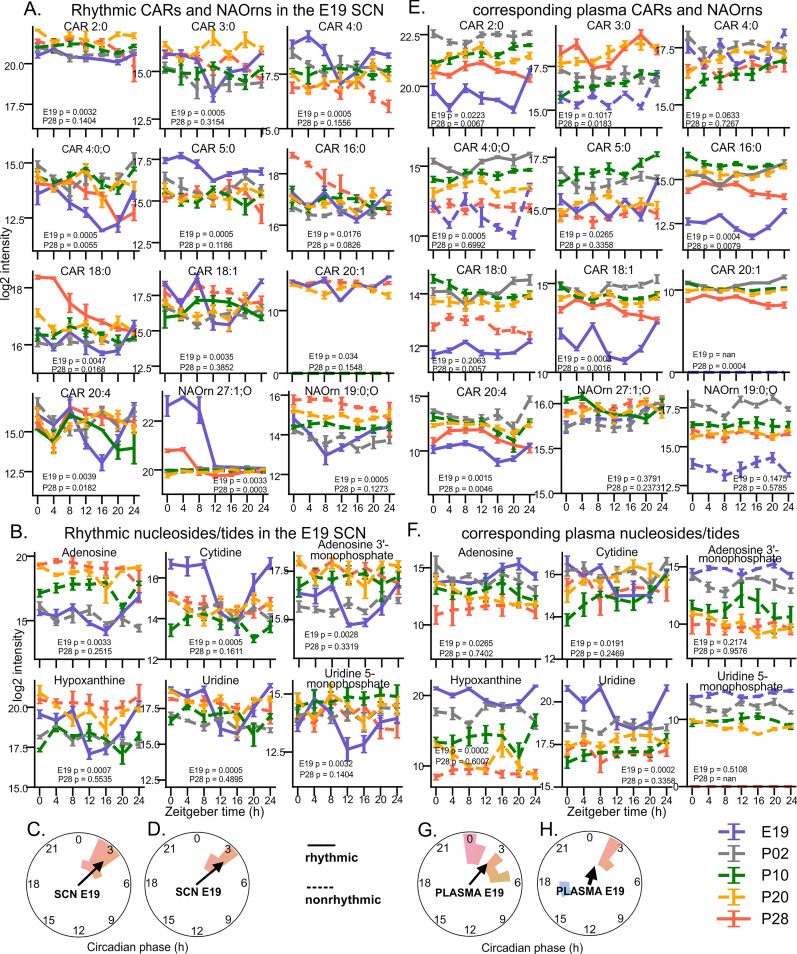
N-Acylornithines (NAOrn), acylcarnitines (CAR), nucleosides, and nucleotides identified as rhythmic at E19 SCN. NAOrns, CARs, and bases in the SCN, with their corresponding profiles in plasma. **(A)** Circadian time profiles of NAOrns and CARs rhythmic in the E19 (blue) SCN, with corresponding traces at P02 (gray), P10 (green), P20 (orange), and P28 (red). **(B)** Circadian time profiles of nucleosides/tides rhythmic in the E19 SCN. **(C)** Polar histogram of the NAOrns and CARs in the E19 SCN, and **(D)** polar histograms of nucleosides/tides in the E19 SCN, with calculated Rayleigh vectors showing the mean phase. Corresponding time profiles **(E, F)** and polar histograms **(G, H)** of the same lipids and nucleosides/tides in plasma. Rhythmicity was determined by eJTK; full or dashed lines depict the profiles that either did or did not pass the significance threshold (FDR-adjusted *P* < 0.05), respectively. Mean ± S.E.M, *n* = 4–5/time point, annotated with eJTK FDR corrected empirical *P* values for E19 and P28 data.

We identified 10 rhythmic carnitines (CARs, with either short acyl chain, such as acetyl-l-carnitine CAR 2:0, or long chain, such as palmitoyl-l-carnitine CAR 16:0, and arachidonoylcarnitine CAR 20:4) and 2 rhythmic long chain N-acylornithines (Fig 6A, NAOrns), clustering around ZT3 in the E19 SCN (Fig 6C), slightly phase-delayed in comparison to their fetal plasma levels (Fig 6E and 6G).

Interestingly, E19 SCN also showed rhythmic levels of four nucleosides, especially cytidine and uridine (Fig 6B), and of two nucleotides (AMP and UMP, Fig 6B). All 4 nucleosides were rhythmic in fetal plasma (Fig 6F) with slightly delayed phase in the fetal SCN (Fig 6D and 6H).

Since all of the above-mentioned compounds lost their rhythmicity after birth, we propose that levels of at least some of them oscillate in the E19 SCN in response to maternal feeding rhythms after being transported across the placenta into fetal plasma.

## Discussion

In search of potential nonhumoral communication between the maternal and fetal SCN, we used a metabolomic and lipidomic approach to identify rhythmic metabolites, combined with RT qPCR to analyze the rhythms in the expression of clock genes and clock/metabolism-regulated genes in the fetal and postnatal SCN and plasma. We used both techniques to detect daily gene expression and metabolomics profiles in the SCN samples collected at the fetal stage (E19) and compared them to 4 postnatal developmental stages (P02, P10, P20, to P28). P02 was selected as an early postnatal stage in which the SCN morphology is still developing, P10 represents a stage in which the morphological development of the SCN is complete, P20 is a developmental stage just before weaning, and P28 is an adult-like developmental stage (reviewed in [13]). The results of this study are consistent with our previous data showing that at E19, the transcript levels of the canonical clock genes in the rat SCN are low (except for *Bmal1*) and their circadian rhythms are negligible or absent, as we demonstrated using various techniques to detect transcript levels, including in situ hybridization [17, 18], RT qPCR [16], and RNA-seq analysis [19].

Comparison of gene expression profiles over the developmental stages revealed distinct patterns of how rhythms in expression of individual clock genes participating in the TTFL develop in the SCN from the fetal to the postnatal stage. The rhythmicity seems to be detectable earlier for genes driven via E-box (*Per2*, *Nr1d1*) than for *Bmal1*. The results also suggest that the development of the *Bmal1* rhythm depends on the mutual phasing of *Nr1d1* and *E4bp4*, i.e., genes negatively regulating expression of *Bmal1* (via RORE and D-box, respectively). The *Bmal1* rhythm first develops only after P02, via increased transcriptional inhibition through higher *Nr1d1* expression during the daytime, lower expression during the nighttime and decreased *E4bp4* levels during the daytime. Importantly, at E19, the rhythm of *E4bp4* expression likely arises from input pathways outside the TTFL because expressions of *Nr1d1* (this data) and *Ror* [19] are not rhythmic at this stage. The amplitudes of the clock gene expression profiles attain the adult-like levels at P10, although output rhythm mediated by *Dbp* seems to further develop until P20.

Previous studies of the adult mouse SCN metabolome [24–26] detected rhythms in 22–134 metabolites (9%–16.3% of all detected compounds), depending on the method. We used a combined metabolomics/lipidomics approach that detected 110 rhythmic compounds in the rat P28 SCN (21.6% of all detected compounds), mostly including various long C-chain lipids, but also metabolites such as piperidine (cyclic secondary amine), ornithine (polyamine precursor), and gluconic acid (potential marker of oxidative stress). As the SCN develops, compounds such as adenosine, AMP and NAA (brain-specific molecule important for myelination and energy metabolism [28]) increase in concentration, while choline, phosphatidylcholines (dietary sources of choline and components of membranes), taurine (sulfonate AA), polyamines (spermidine), or specific FAs (such as omega-3 precursor tetracosapentaenoic acid FA 24:5) decrease in concentration, reflecting the requirements of neuronal differentiation and the general developmental trends in the whole brain [29, 30].

In our study, examination of the correlation between individual compounds highlights the two major developmental turning points—birth and weaning—at which the metabolism of the SCN shifts abruptly due to a change in nutrition, and the temporal coherence of the metabolome and lipidome decreases dramatically. The dynamic of the postnatal transition from the breastfeeding to solid food consumption is gradual and has been reviewed previously [31, 32]. Briefly, after birth, rat pups nurse almost exclusively during the light phase when their mothers are resting. Natural weaning begins after the pups are sighted at P14 and progresses gradually till P30 with the accelerated speed between P20 and P30. At P28, the proportion of food consumed during darkness approaches that of adult rats [31, 33]. The exclusively nocturnal consumption of solid food after weaning decreases the overall correlation between lipid classes but also quickly establishes circadian rhythms in metabolism.

Interestingly, the rhythms of clock gene expression in the SCN are well developed already by P10, whereas the regulation of metabolite levels in the SCN is almost arrhythmic until weaning is completed. One possible reason for the slower development of local metabolic rhythms may be due to a conflict between the phases of energy metabolism in peripheral tissues and in the SCN. Indeed, circadian rhythms in metabolic organs such as the liver are aligned with milk consumption during the day when the mother is resting, and during weaning, the phase gradually reverses due to solid food consumption during the night [33]. In contrast, the phase of clock gene expression in the SCN remains the same throughout development (Fig 1 and [17, 18]).

Previously, it has been shown that phase of the developing fetal clock is set prenatally by maternal signals, before the robust rhythms in clock gene expression develop in the fetal SCN [21, 34]. However, the mechanism of maternal synchronization is only partially understood and mainly humoral signals have been investigated (reviewed in [20, 35]). In particular, a role of the hormone melatonin has been demonstrated in rats [36], but the results do not explain maternal synchronization in melatonin-deficient rodent species, such as the commonly used mouse strains. More recently, a role of glucocorticoids has also been considered [37, 38]. Maternal body temperature rhythm has not been systematically studied as a signal entraining the fetal SCN likely because amplitude of the body temperature rhythm during pregnancy decreases [39]. Nevertheless, ambient temperature changes are capable of resetting the neonatal rat SCN [40]. The maternal feeding/fasting rhythm may represent a powerful signal for the fetal SCN [41], the mechanism of which has not yet been investigated. The role of the signal is supported by the results of this study documenting the earlier appearance of *E4bp4* rhythm in the fetal SCN that may result from responses to rhythmic signals outside the TTFL and potentially contribute to setting the initial phase of the developing clock. Indeed, *E4bp4* expression is not only controlled by the circadian clock but also responds to various external rhythmic signals [42, 43], including feeding/fasting rhythms in peripheral tissues [44]. The signals driving the *E4bp4* rhythm likely originate from the maternal body, as the rhythm was lost in their absence after birth (at P02). Nevertheless, direct evidence that *E4bp4* is one of the genes sensitive to maternal signals in the fetal SCN remains to be provided by pharmacological inhibition of specific transporters or by targeted silencing of *E4bp4*.

To identify the rhythmic signals, we analyzed polar metabolites and complex lipids that are rhythmic in the fetal SCN when the circadian clock has not yet started to operate at the tissue level, and compared these profiles with those at the postnatal stages. Using the approach, we were able to identify rhythmic polar metabolites and lipids in the same SCN sample [45]. The results confirmed that the rat fetal SCN at E19 is metabolically rhythmic, as previously shown by the 2-deoxy glucose marker [21], but the rhythms are most likely not the output of the fetal clock. Interestingly, a rhythm of glucose uptake has been demonstrated in undifferentiated stem cells, which do not possess a functional circadian clock [46].

To identify potential novel maternal signals to the fetal SCN, we focused on the levels of compounds of dietary origin, such as EAA and dietary lipids, which may serve as maternal feeding/fasting signals to the fetal clock. To reduce the likelihood of errors in detecting the rhythmic targets [47] in the E19 SCN, we verified them using the differential rhythmicity analysis method CircaCompare [48], which shows that in addition to passing the eJTK significance threshold, all of these metabolites, with the sole exception of CAR 3:0, differ significantly in at least one of the circadian parameters (mesor, amplitude and/or phase; see S1 Data). We found circadian rhythms in many polar metabolites in the fetal SCN. There are 9 EAA and 6 conditionally essential AA [49, 50], and at least 4 of them (methionine, phenylalanine, proline, and threonine) display rhythms in the adult plasma, fetal plasma, and the E19 SCN. All of the EAA and conditionally EAA that were rhythmic in the fetal plasma and fetal SCN are actively conveyed by placental transporters [51] that are rhythmic in alignment with the maternal feeding/fasting regime (SLC7A5, SLC38A1, or SLC38A2) [52, 53]. The EAA and conditionally EAA that were identified as rhythmic in the fetal SCN may potentially play multiple roles, including (1) regulation of proteosynthesis-methionine is the universal start codon (AUG) in protein translation and leucine is a key regulator of the mechanistic target of rapamycin (mTOR) pathway [54], which controls protein synthesis and cell growth, (2) posttranslational and epigenetic modifications (methionine, histidine), (3) neurotransmission-phenylalanine is a precursor for dopamine that was shown to affect the fetal SCN [55], and (4) energy metabolism (leucine, proline, and methionine).

The analysis of lipid compounds revealed rhythms in the levels of 10 carnitines (CAR) and two N-acylornithines (NAOrn 27:1 and NAOrn 19:0) in the fetal SCN with high levels during the daytime. All of these lipids were highly enriched in the fetal SCN compared to fetal plasma, showing their importance for brain function. CARs are the cofactors for beta-oxidation, shuttling FAs to mitochondria. In adults, they are released by the liver in response to fasting and serve as an alternative energy source for the brain [56]. During development, maternofetal transport of carnitines is considered important in preparing the fetus for its lipid-rich postnatal milk diet [56], though the fetus can also synthesize CAR when the maternal carnitine supply is limited [57]. NAOrns belong to the fatty acylamines, which serve as intermediates between the metabolism of AA and FAs. NAOrn 27:1 exhibited a robust rhythm that disappeared immediately after birth and only reappeared in adulthood (P28), albeit with significantly lower amplitude. It could act as a signaling molecule within the cells and influence physiological processes. Some N-acylamino acids are of dietary origin, but NAOrn 27:1 has not been studied, so its endogenous origin cannot be ruled out.

Other potentially food-derived compounds [58] actively transported via placenta are nucleosides and nucleotides [59]. The levels of hypoxanthine (intermediate in adenosine metabolism), adenosine (plus adenosine 3′-monophosphate), cytidine (a precursor of uridine), and uridine (plus uridine 5-monophosphate) exhibited synchronized circadian rhythms in the fetal SCN with minimal levels at the beginning of night (around ZT12–16). Interestingly, levels of cytidine were the highest in the SCN at E19 and declined immediately after birth (P02). Apart from the role as a pyrimidine component of RNA, cytidine has been considered to participate in the control of neuronal-glial glutamate cycling in order to reverse excess glutamate neurotransmission [60, 61], as well as in phospholipid metabolism and membrane stability [62–64]. The food intake-related rhythm in cytidine levels in the fetal SCN may thus facilitate the intracellular and extracellular processes and synaptic neurotransmission on neuronal membranes.

Based on the results of this study, we propose that nonhumoral molecules of maternal origin are transported into the fetal blood and reach the developing SCN, where their rhythmic variations may affect the fetal SCN and potentially contribute to maternal entrainment of the developing clock. Indeed, our data suggest that the fetal SCN receives multiple food-related rhythmic signals from the mother via daily rhythm in the levels of EAA (and conditionally-EAA) and dietary lipids that can rhythmically regulate cellular processes, including protein synthesis, neurotransmitter synthesis, and energy metabolism, in the absence of the functional fetal SCN clock. At least some of these rhythmic processes can potentially participate in setting the phase of the developing fetal clock.

### Limitations

It is important to note, that in the SCN, the rhythms were detected at the tissue level, and their absence or presence may be due to processes at the level of individual cells as well as at the level of cell populations. Due to very low amplitudes of the gene expression rhythms during the fetal stage, the assignment of the emerging rhythmicity is highly dependent on the precise assessment of the developmental stage with a half-day resolution, the detection method and the stringency of the threshold for significance of the rhythm. This may justify inconsistencies in the interpretation of results between various studies. Another potential limitation is comparing data for plasma from the adult male rats (P28) with the fetal plasma (E19). It is possible that the lipidomics and metabolomics profiles would be different in pregnant rats. However, data from P28 plasma are not decisive for interpreting the main findings on the development of the profiles in the SCN, as the dietary origin of these compounds is documented. Finally, to demonstrate a causal relationship between maternal rhythms and the fetal circadian metabolome, future experiments should examine its development under conditions where (1) the maternal feeding regime is disrupted or (2) the fetal clock develops under arrhythmic conditions, as in the case of genetic mouse models of *Bmal1* knockout mothers crossed with wild-type fathers.

## Materials and methods

### Ethics statement

Experiments are performed in accordance with a valid experimental project reference number AVCR 8271/2022 SOV II. The project is approved by the Animal Care and Use Committee of the Institute of Physiology of the Czech Academy of Sciences, as well as by the Resort Professional Commission of the CAS for Approval of Projects of Experiments on Animals. The animals are housed in facilities accredited by the Czech Ministry of Agriculture. Experiments are carried out under veterinary supervision, complying with Act No. 246/1992 Coll. and Decree No. 419/2012 Coll., implementing Directive 2010/63/EU of the European Parliament and of the Council regarding the protection of animals used for scientific purposes. The 3Rs principles are applied to the maximum extent possible.

### Animals

Adult Wistar:Han rats (Institute of Physiology of the Czech Academy of Sciences) were kept at 21 ± 2 °C with free access to food and water at the 12-hour light/12-hour dark cycle (the lights were switched on at 06:00, which was designated as Zeitgeber time 0, ZT0, and switched off at 18:00, which was designated as ZT12). The light was provided by overhead 40 W fluorescent tubes (illumination level of ~150 lux depending on cage position in the animal room). Female and male rats were mated for one night and the vaginal smears were performed on the next morning. The sperm-positive rats were housed individually. Fetuses (*n* = 70) were collected at embryonic day (E)19. Pups (*n* = 280) were left with their mothers after delivery, and euthanized at postnatal days (P)02, P10, P20, and P28 (*n* = 70 per each age). At each developmental stage, 5 (in rare cases 3–4) brains and plasma from fetuses or pups were collected every 4 hour during the 24-hour cycle (corresponding to ZT0, ZT4, ZT8, ZT12, ZT16, ZT20, and ZT24). For ZT0 and ZT12, the animals were sacrificed immediately after the lights were switched on at 6:00 and off at 18:00, respectively. At P10, samples collected for analysis at ZT24 are missing. In case of the fetal samples, each litter was divided into two groups of fetuses and each was used for one of the methods. For RT-qPCR, brains were frozen on dry ice and stored at −80 °C until the SCN samples were obtained with laser-capture microdissection as described previously [16]. For liquid chromatography–mass spectrometry (LC–MS), plasma was obtained from blood collected by decapitation of fetuses or pups, and SCN were isolated from the brain slices by a precooled microbiopsy punch, resulting in a cylindrical sample of SCN-containing frozen tissue with a 0.3 mm diameter and approximately 0.4 mm height, and the samples were stored at −80 °C until further processing and analysis.

### RT-qPCR

We detected mRNA levels of clock genes (*Bmal1, Per2*, **Nr1d1/Rev-Erb*α)* and clock- and metabolism-related genes *(Dbp, E4bp4/Nfil3*) in the SCN at 5 developmental stages (3–5 replicates × time point × group). RNA was isolated using an RNeasy Micro kit (Qiagen), and up to 0.5 µg was then reverse-transcribed into cDNA using a High-Capacity cDNA RT Kit (ThermoFisher). Diluted cDNA was then amplified on LightCycler480 (Roche) using SYBR Select qPCR Master Mix (ThermoFisher) as described previously [65]. Relative quantity was calculated using the Livak’s ΔΔCt method against the reference gene *Tbp*. For each gene, all samples were analyzed in the same RT-qPCR run. The amplitude and phase of the clock genes were calculated by cosinor as described previously [66]—briefly, RT-qPCR data were fitted with cosine curves defined by the equation Y = Mesor + (Amplitude × cos(2 × pi × (X-Acrophase)/Wavelength)) using the least-squares regression method implemented in Prism 7 (GraphPad). For the list of primers, see S2 Data.

### LC–MS-based metabolomics

Lipids and metabolites were extracted using bi-phase extraction with methanol, methyl *tert*-butyl ether, and 10% water. Lipidomics and metabolomics were performed on an LC–MS system consisting of a Vanquish UHPLC System (Thermo Fisher Scientific, Bremen, Germany) coupled to a Q Exactive Plus mass spectrometer (Thermo Fisher Scientific, Bremen, Germany) [45], followed by processing of raw instrumental files using MS-DIAL [67]. For a detailed description of the extraction, LC–MS parameters, quality control, and data processing, see [68, 69]. Absolute values (peak heights) of all identified compounds were further analyzed; no outliers were removed. The raw data are presented as a part of the multi-tissue Circadian Ontogenetic Metabolomics Atlas (COMA), which is publicly available (https://coma.metabolomics.fgu.cas.cz) and serves as an interactive and reusable resource for future metabolomics studies, facilitating deeper insights into circadian rhythms and metabolic processes.

### Data processing and statistical analysis

Median imputation was used for metabolomics data in case ≤2 replicates out of five/time point were missing and the total missing values were ≤20%/metabolite. Differences in average levels between metabolites in both groups were analyzed by DESeq2 using RNAlysis 4 GUI [70]. To identify potentially rhythmic compounds and expressed genes, one-way ANOVA was used to select those with significant (*P* < 0.05) variation across time points, followed by the rhythmicity detection algorithm eJTK (https://biodare2.ed.ac.uk/) [71]. The rhythmicity threshold was set as (Benjamini–Hochberg false discovery rate-adjusted) empirical *P* < 0.05. To verify the rhythmic targets, the differential rhythmicity analysis method CircaCompare [48] was used to compare mesor, amplitude, and phase between E19 and P28 SCN and plasma profiles, with the threshold for differential rhythmicity set to Benjamini–Hochberg false discovery rate-adjusted *P* < 0.05.

For comparisons, the amplitude was calculated for all compounds, even those that did not pass the threshold of significant rhythmicity. Rhythmic z-score normalized compounds in each group were clustered according to their phase determined by eJTK using gap-statistics K-means clustering in RNAlysis 4. All z-score normalized compounds were used to calculate Spearman’s rank correlation and principal component analysis (PCA). An overrepresentation analysis of complex lipids and polar metabolites enriched in the rhythmic groups against the background of all 851 identified metabolites was performed using LORA [27] and WEB-based GEne SeT AnaLysis Toolkit (https://www.webgestalt.org), respectively. Plots were prepared in either Prism 8 (Graphpad, USA), or seaborn + matplotlib, and statistical comparisons (one-way and two-way ANOVA, Mann–Whitney test, and *t* test) were performed in Prism, or Python packages scipy and statsmodels.

## Supporting information

S1 Fig Metabolites with increasing levels during the development. Temporal profiles of polar metabolites and lipids with SCN levels significantly increasing from E19 to P28. Rhythmicity was determined by eJTK; full or dashed lines depict the profiles that either did or did not pass the significance threshold (FDR-adjusted *P* < 0.05), respectively.
(TIF)

S2 Fig Metabolites with decreasing levels during the development. Temporal profiles of polar metabolites and lipids with SCN levels significantly decreasing from E19 to P28. Rhythmicity was determined by eJTK; full or dashed lines depict the profiles that either did or did not pass the significance threshold (FDR-adjusted *P* < 0.05), respectively.
(TIF)

S3 Fig Metabolites detected at specific developmental stages. Temporal profiles of polar metabolites and lipids detected in the SCN at E19 (prenatal), E19–P02 (perinatal), P02–P28 (postnatal), P02–P20 (until weaning), or P28 (after weaning). Rhythmicity was determined by eJTK; full or dashed lines depict the profiles that either did or did not pass the significance threshold (FDR-adjusted *P* < 0.05), respectively.
(TIF)

S4 Fig Lipid tree. Lipid tree generated by LORA (https://lora.metabolomics.fgu.cas.cz/)-hierarchical circular dendrogram that organizes lipid categories (inner circle labels) and their structurally related classes (represented by the branching structure leading to the outer circle segments) based on their shared chemical features. It shows each lipid annotated as specific level (from the center to the rim: category—class—species—molecular species—SN position—defined structure—full structure—complete structure) according to Goslin levels [72] and SHORTHAND2020 classification system (https://apps.lifs-tools.org/goslin/). Nomenclature: SM, sphingomyelins, TG, triacylglycerols, CAR, acylcarnitines, CL, cardiolipins, CER, ceramides, DG, diacylglycerols, FA, fatty acyls, HexCer, hexosylceramides, LPC, lysophosphatidylcholines, LPE, lysophosphatidylethanolamines, LPI, lysophosphatidylinositol, LPG, lysophosphatidyglycerols, PC, phosphatidylcholines, PCe, ether phosphatidylcholines, PE, phosphatidylethanolamines, PEe, ether phosphatidylethanolamines, PG, phosphatidyliglycerols, PI, phosphatidylinositols, PS, phosphatidylserines, FAHFA, fatty acyl esters of hydroxy fatty acids, NAE, N-acylethanolamines, GL, glycerolipids, ST, sterols, GP, glycerophospholipids, GL, glycerolipids, SP, sphingolipids.
(TIF)

S1 Data Dataset. (CSV)

S2 Data List of primers. (DOCX)

## Abbreviation

AAs: amino acids
CAR: carnitines
COMA: Circadian Ontogenetic Metabolomics Atlas
EAA: essential amino acids
FA: fatty acids
GP: glycerophospholipids
HILIC-MS: hydrophilic interaction chromatography-mass spectrometry
mTOR: mechanistic target of rapamycin
ORA: over-representation analysis
PCA: principal component analysis
RPLC–MS: liquid chromatography–mass spectrometry
SCN: suprachiasmatic nuclei
TTFL: transcriptional and translational feedback loop
