# Supplementary figures and images for: Maternal food-derived signals oscillate in the fetal suprachiasmatic nucleus before its circadian clock develops
Source: PLoS Biol. 2025 Sep 26;23(9):e3003404. doi: 10.1371/journal.pbio.3003404 (PMC12469392; doi:10.1371/journal.pbio.3003404)

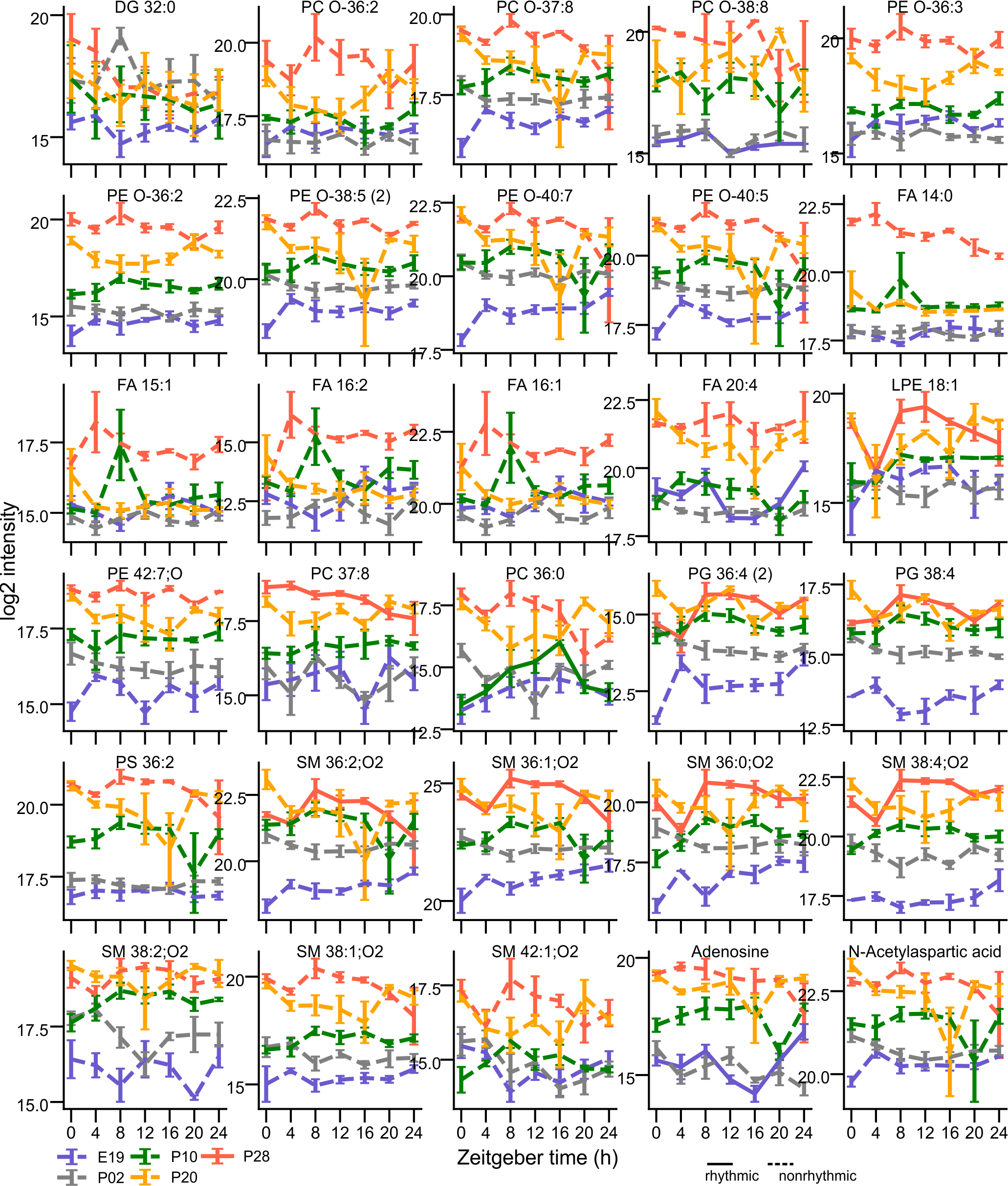

Supplement: S1 Fig — Temporal profiles of polar metabolites and lipids with SCN levels significantly increasing from E19 to P28. Rhythmicity was determined by eJTK; full or dashed lines depict the profiles that either did or did not pass the significance threshold (FDR-adjusted P < 0.05), respectively. (TIF) [file pbio.3003404.s001.tif]

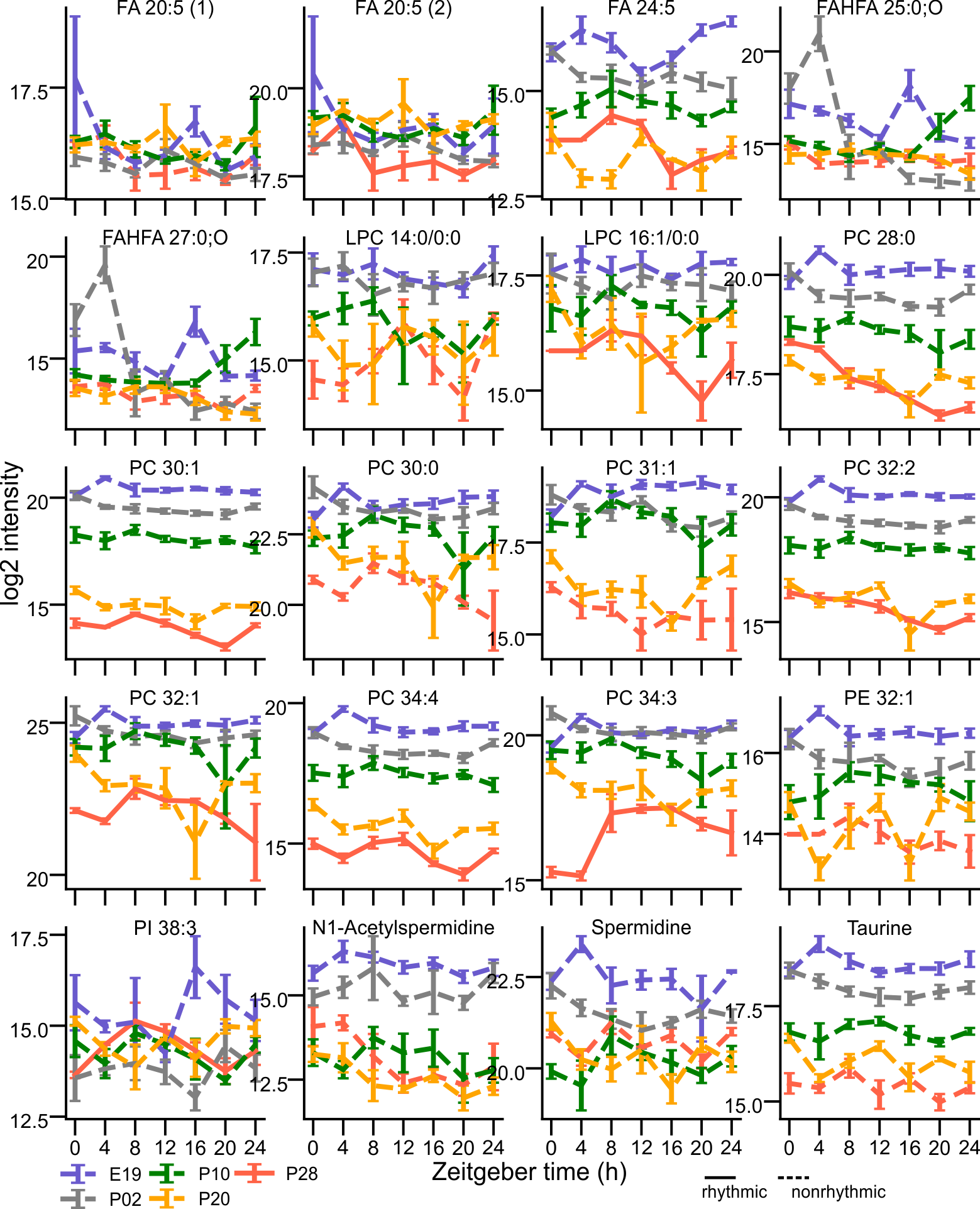

Supplement: S2 Fig — Temporal profiles of polar metabolites and lipids with SCN levels significantly decreasing from E19 to P28. Rhythmicity was determined by eJTK; full or dashed lines depict the profiles that either did or did not pass the significance threshold (FDR-adjusted P < 0.05), respectively. (TIF) [file pbio.3003404.s002.tif]

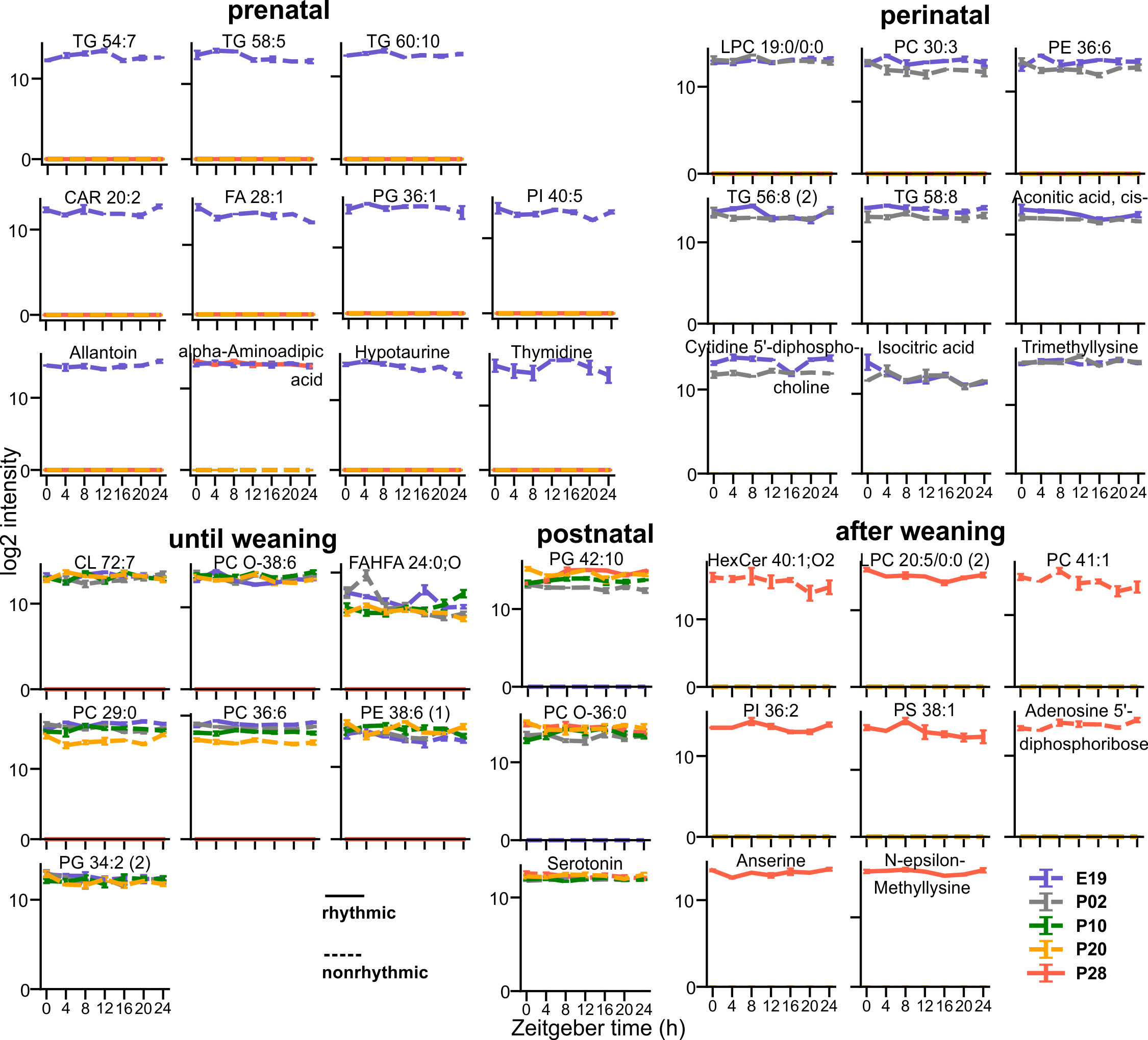

Supplement: S3 Fig — Temporal profiles of polar metabolites and lipids detected in the SCN at E19 (prenatal), E19–P02 (perinatal), P02–P28 (postnatal), P02–P20 (until weaning), or P28 (after weaning). Rhythmicity was determined by eJTK; full or dashed lines depict the profiles that either did or did not pass the significance threshold (FDR-adjusted P < 0.05), respectively. (TIF) [file pbio.3003404.s003.tif]

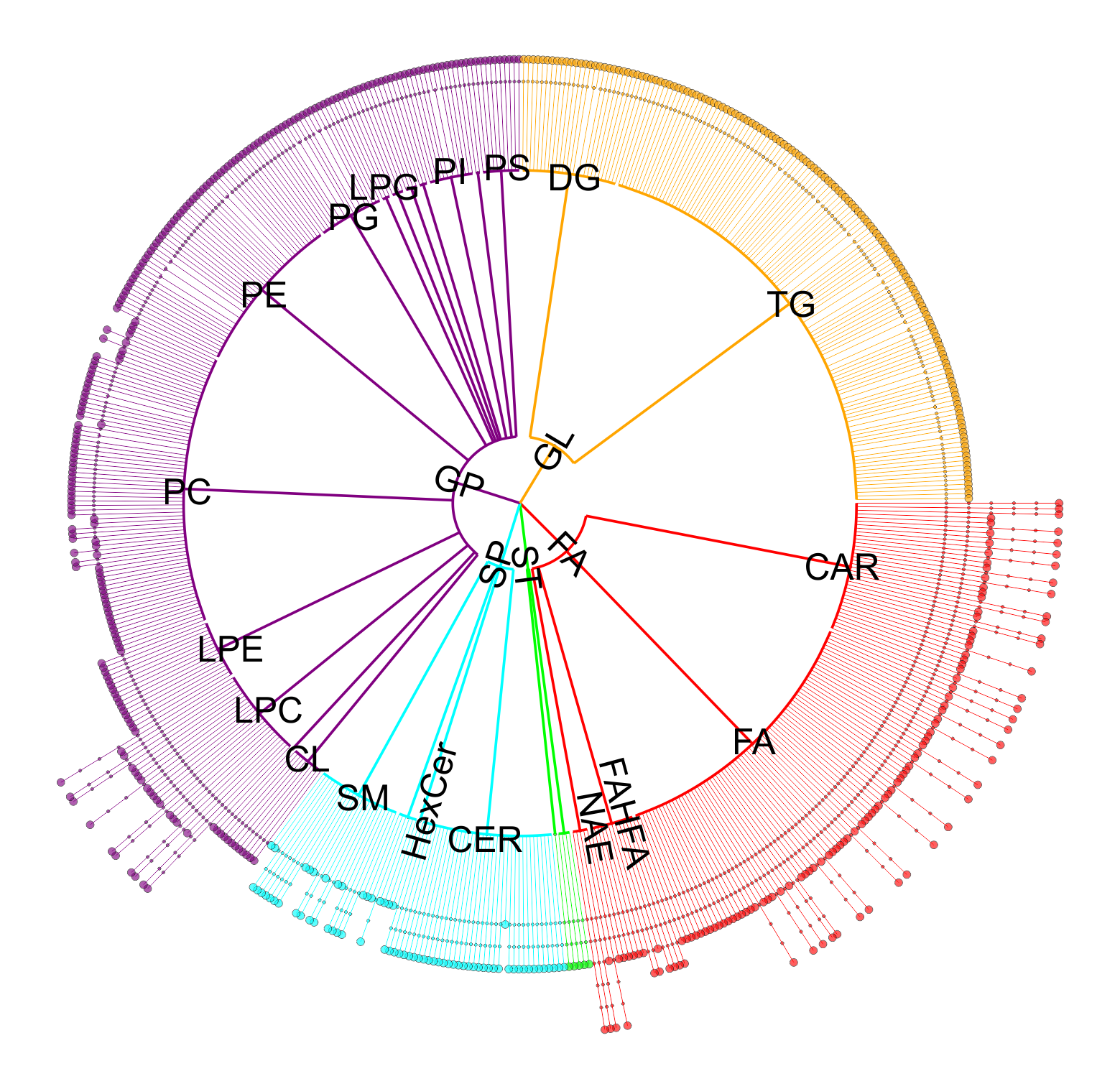

Supplement: S4 Fig — Lipid tree generated by LORA (https://lora.metabolomics.fgu.cas.cz/)-hierarchical circular dendrogram that organizes lipid categories (inner circle labels) and their structurally related classes (represented by the branching structure leading to the outer circle segments) based on their shared chemical features. It shows each lipid annotated as specific level (from the center to the rim: category—class—species—molecular species—SN position—defined structure—full structure—complete structure) according to Goslin levels [72] and SHORTHAND2020 classification system (https://apps.lifs-tools.org/goslin/). Nomenclature: SM, sphingomyelins, TG, triacylglycerols, CAR, acylcarnitines, CL, cardiolipins, CER, ceramides, DG, diacylglycerols, FA, fatty acyls, HexCer, hexosylceramides, LPC, lysophosphatidylcholines, LPE, lysophosphatidylethanolamines, LPI, lysophosphatidylinositol, LPG, lysophosphatidyglycerols, PC, phosphatidylcholines, PCe, ether phosphatidylcholines, PE, phosphatidylethanolamines, PEe, ether phosphatidylethanolamines, PG, phosphatidyliglycerols, PI, phosphatidylinositols, PS, phosphatidylserines, FAHFA, fatty acyl esters of hydroxy fatty acids, NAE, N-acylethanolamines, GL, glycerolipids, ST, sterols, GP, glycerophospholipids, GL, glycerolipids, SP, sphingolipids. (TIF) [file pbio.3003404.s004.tif]
