# Supplementary material for: Maternal food-derived signals oscillate in the fetal suprachiasmatic nucleus before its circadian clock develops
Source: PLoS Biol. 2025 Sep 26;23(9):e3003404. doi: 10.1371/journal.pbio.3003404 (PMC12469392; doi:10.1371/journal.pbio.3003404)
Supplement: S2 Data — (DOCX) [file pbio.3003404.s006.docx]

**Supplementary Table S2**. Sequences of primers for rat genes used for RT-qPCR.

| **Gene** | **Forward and Reverse Primer Sequence** |
| --- | --- |
| *Tbp* | Forw: CATCATGAGAATAAGAGAGCC Rev: GGATTGTTCTTCACTCTTGG |
| *Per2* | Forw: GAATTTTCACAACAACCCAC Rev: TGTAGGATCTTCTTGTGGATG |
| *Bmal1 (Arntl)* | Forw: ATGAAAACATTGAGAGGTGC Rev: GGATCTTGAAGACAGATTCG |
| *Rev-Erbα (Nr1d1)* | Forw: GCTGTGCGGGAGGTGGTAGAAT Rev: TGTAGGTTGTGCGGCTCAGGAA |
| *Dbp* | Forw: GCTAATGACCTTTGAACCTG Rev: AGTACTTCTCATCCTTCTGTTC |
| *E4bp4 (Nfil3)* | Forw: GCAGGAGCCCGTGGAGTTGGAGAG  Rev: AGGAGGGGAGGGGAGTGGGAGTAGGT |
